# FamCASP—A Support Model for Individualised Support for Family Members in Routine Cancer Care: Development and Validation

**DOI:** 10.1111/scs.70164

**Published:** 2025-12-03

**Authors:** Maria Samuelsson

**Affiliations:** ^1^ Department of Care Science Malmö University, Faculty of Health and Society Malmö Sweden

**Keywords:** cancer, caregivers, co‐design, development, family members, intervention, support

## Abstract

**Introduction:**

A cancer diagnosis may have negative consequences on family members' health and well‐being. Although support for family members has been stressed for decades, family members continue to report unmet needs for support. Suggested explanations are the lack of individualisation and the lack of implementation. Therefore, the overall aim of this project is to develop and evaluate a support model for individualised support in routine cancer care. The objective of this paper is to describe the support model's development and validation.

**Method:**

To address the complex phenomenon of offering individualised support for family members in routine cancer care, we applied a dynamic multi‐method approach, guided by the Medical Research Council's Framework for Development and Evaluation of Complex Interventions. The support model was developed with healthcare professionals, family members, and stakeholders to ensure relevance and applicability. The development and validation involved a literature review, qualitative interviews, and repeated consultations. Ethical approval and written informed consent were obtained.

**Results:**

The FamCASP support model is a nurse‐led, interprofessional model for structured yet individualised support for family members to prevent cancer‐related illnesses in routine cancer care. It consists of two parts—one generic model and diagnosis‐specific modules—and is adaptable to local contexts and allows for the integration of existing support.

**Limitations:**

Throughout the support model development, local (national) fit was emphasised, which may limit international application. Further, to this date, only one diagnose‐specific module has been developed (colorectal cancer). Hence, the applicability of the support model in other contexts needs further exploration.

**Conclusion:**

Through concurrent consideration of family members' needs and prerequisites for support in routine care, a tailored support model was developed and assessed as relevant, usable and implementable by healthcare professionals, family members and stakeholders. Evaluations of feasibility and effectiveness are warranted prior to implementation.

## 1 Background

The increase in cancer incidence exerts physical, emotional and financial strain on families, communities and healthcare systems [1], and the consequences of cancer on family members' health aggravate the strain. Family members may suffer from impaired psychological [2] as well as physical health, for example, ischaemic heart disease [3], coronary heart disease, and stroke [4], risks that have been shown to persist over time. This may weaken family members' capacity to support the patient, a support that has proved significant for survival rates [5, 6]. In addition, ill health among family members may result in a decreased capacity to maintain working life and may lead to more family members engaging in caregiving [7, 8, 9]. Consequently, preventive support for family members is stressed in research [10, 11] and standards for clinical cancer care [12, 13, 14].

Despite the evidence of the impact of cancer on family members' health and the need for support, family members continue to report unmet supportive care needs [15, 16, 17, 18, 19] and to have high rates of ill health [4]. This motivates further study of support for family members and how this can be offered [20]. Due to the ongoing transformation of cancer care, from in‐hospital to outpatient care [21, 22, 23], the outpatient care setting is recommended for support initiatives for family members. Therefore, our project focused on psychosocial support in the routine outpatient cancer care setting. For this project, no distinction is made between family caregivers and family members since these concepts are used interchangeably in the literature [24]. Thus, family members were invited on a relational basis in accordance with the definition by Wright and Leahey [25]: ‘the family is who they say they are’ (p. 55), which involves biological families, next of kin, and/or friends. Previous research highlights that the supportive care needs of family members and of persons diagnosed with cancer are not always consistent [19], which is why this project focuses solely on the family members' perspectives instead of applying a whole family approach and, with regard to the specific needs of minor children [26], only on family members > 18 years old.

Support for family members in routine cancer care is a complex phenomenon in a complex setting. On the one hand, family members need support tailored to their individual needs [27, 28, 29]; on the other hand, cancer specialist nurses, who are assigned to support family members in outpatient care, need to balance a range of responsibilities, legislations, and interprofessional collaborations [30, 31]. Previous support interventions have been criticised for not sufficiently considering the clinical context, the support intervention therefore being unimplementable [21, 32]. Thus, promising support models might fail to reach the larger population outside of study participants. However, support models applicable in real‐world care settings tend to solely focus on family members as caregivers [33], and thus risk failing to cover other psychosocial needs family members may have, for example, emotional, existential or relational needs. In addition, they tend to be of universal design and thus not tailored to individual needs. Hence, we developed a support model for individualised support in routine care with the overall aim of preventing negative health outcomes among family members: the FamCASP (Families experiencing Cancer—Support to Prevent cancer‐related illness). The development was conducted with a simultaneous focus on both prerequisites for support in the intended context and family members' supportive care needs. The aim of this paper is to describe the support model development and validation.

## 2 Design

**FIGURE 1:**
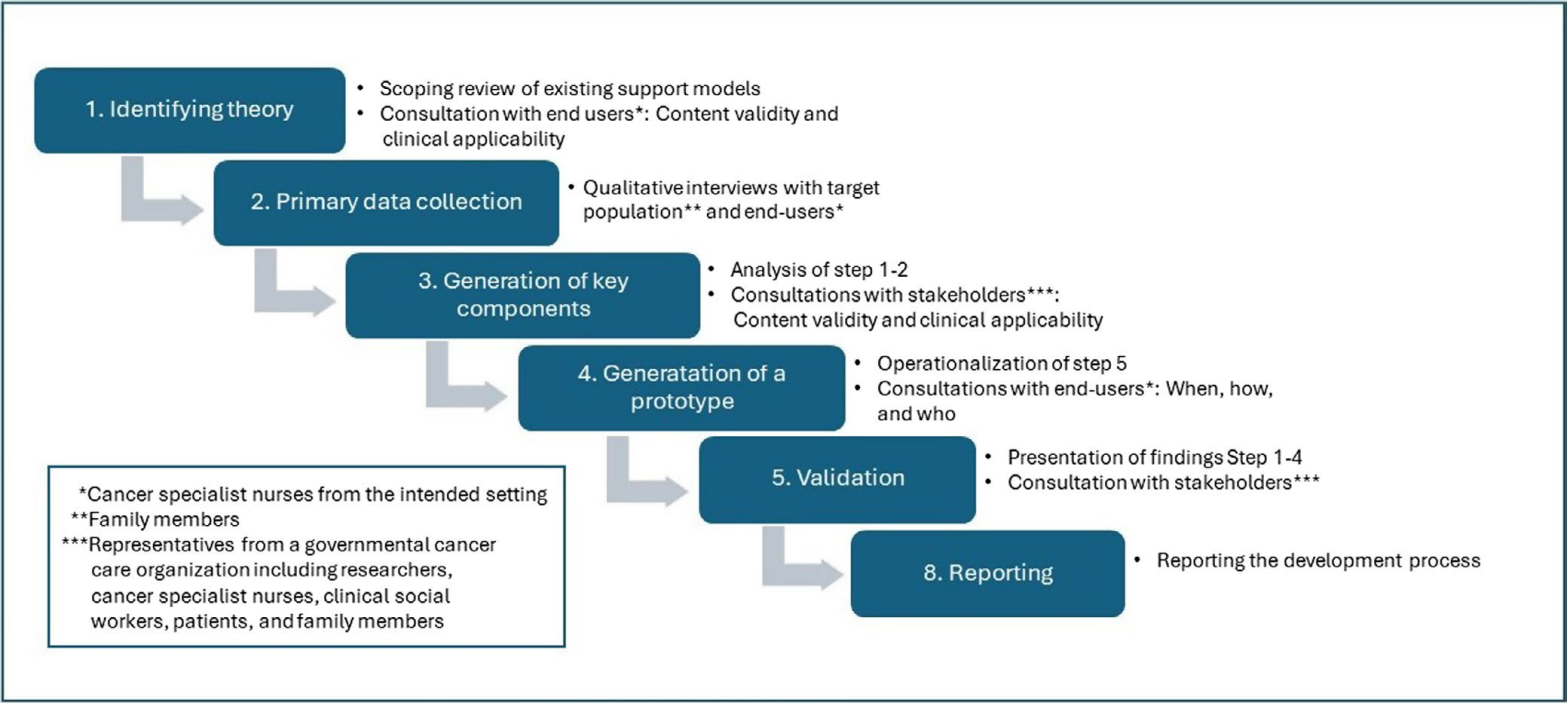
Overview of the process of development.

The support model development applied an iterative multi‐method approach guided by the Medical Research Council's (MRC) Framework for Development and Evaluation of Complex Interventions [34, 35, 36], since they offer guidance on the development of complex interventions that are implementable and lasting in real‐world settings. The development was undertaken in six steps: (Step 1) Identifying theory, (Step 2) Primary data collection, (Step 3) Generation of key components, (Step 4) Generation of a prototype, (Step 5) Validation, and (Step 6) Reporting (this paper). An overview of the study design and the actions is presented in Figure 1. The Transactional Model of Stress and Coping by Lazarus and Folkman [29] was used as a framework for the support model. Thus, the support aims to enhance the family members' coping abilities in order to prevent stress‐related illnesses derived from their cancer experience.

To enhance relevance, usability and uptake [37], the support model development was undertaken in collaboration with end users (cancer specialist nurses), members of the target population (family members) and stakeholders from a governmental cancer care organisation involving cancer specialist nurses, researchers, clinical social workers, patients and family members. Throughout the research process, from design to report, the involved studies were conducted considering the Declaration of Helsinki [38] and with respect for the four moral principles of autonomy, non‐maleficence, beneficence and justice, as described by Beauchamp and Childress [39]. The project was approved by the National Ethical Review Board (Reg. no. 2020–04081; 2021–02601; 2022–02144‐02).

### 2.1 The Swedish Setting

This project is situated in the context of routine outpatient cancer care in Sweden. In Sweden, all persons diagnosed with cancer are entitled to a so‐called Contact Nurse. A Contact Nurse is a cancer specialist nurse who coordinates patient care and also has the responsibility to guide, and offer psychosocial support for, the patient and family members across the cancer trajectory. The frequency and type of contacts (physical or telephone meetings) between the nurse and the patient differ between clinics. Physical contacts between the cancer specialist nurse and family members mainly occur at the time of diagnosis.

## 3 Methods

Data collection involved systematic searches of literature (Step 1), qualitative interviews (Step 2) and consultations (Step 1, 3–5). The steps built on each other and evolved throughout the process in parallel with a continuous moving back and forth between the steps with the purpose of identifying a support model that was relevant for family members' needs as well as implementable in routine care.

### 3.1 Participants

*Step 1: Identifying theory*. Cancer specialist nurses (*n* = 5) caring for persons diagnosed with gastrointestinal, urological, and breast cancers were invited via a cancer specialist nursing education.

*Step 2: Primary data collection*. Colorectal cancer specialist nurses (*n* = 21) and family members (*n* = 23) of persons diagnosed with colorectal cancer. All were women, aged 33–63 (mean 52) and with 7–36 years of experience as registered nurses (mean 22 years). Family members were adult children (*n* = 9) and partners (*n* = 14), aged 29–85 (mean 57). Cancer specialist nurses were invited via the head of department and family members via a gatekeeper (not involved in the project).

*Step 3: Generation of key components*. Stakeholders from a governmental organisation for cancer care: researchers, cancer specialist nurses, clinical social workers, patients, and family members, participated together in three consultations. The mean number of stakeholders per consultation was 10. Participants varied between the consultations, and they were invited during network meetings.

*Step 4: Generation of a prototype*. Cancer specialist nurses (*n* = 4) from the intended setting. Participants were invited via participants in Step 2.

*Step 5: Validation*. Stakeholders from a governmental organisation for cancer care: researchers, cancer specialist nurses, clinical social workers, patients and family members. Participants were invited during a network meeting.

### 3.2 Step 1: Identifying Theory

In Step 1, a scoping review was undertaken, following the methodological framework outlined by Arksey and O'Malley [40], refined by Levac et al. [41] and Colquhoun et al. [42] and described by the Joanna Briggs Institute [43]. The search strategy and the selection process are described in detail elsewhere [33]. A total of 39 support models for adult family members affected by cancer were identified. These were analysed deductively, using semantic thematic analysis by Braun and Clark [44], into type of support model (psychoeducation, psychological and caregiver training) and they were then contrasted against family members' reported supportive care needs as reported in the literature. Across support models, consideration of their clinical applicability was not reported as part of their development. Support offered in routine care only targeted the caregiving needs of family members. Hence, this support did not address emotional needs, nor the phenomenon of family members suppressing their needs. In addition, most were not tailored to individual needs and only two reported considerations of phase‐specific needs. Then, the support models were assessed verbally by end users for content relevance, usefulness and clinical applicability. Comprehensive support models focusing on information and emotional support were assessed as content‐relevant yet not implementable in routine care due to the extent of resources and user knowledge required. Consequently, a need for developing a support model for individualised support was identified, a support model that is sensitive to the fact that needs may shift across the trajectory and that is still applicable in routine care.

### 3.3 Step 2: Primary Data Collection

**FIGURE 2:**
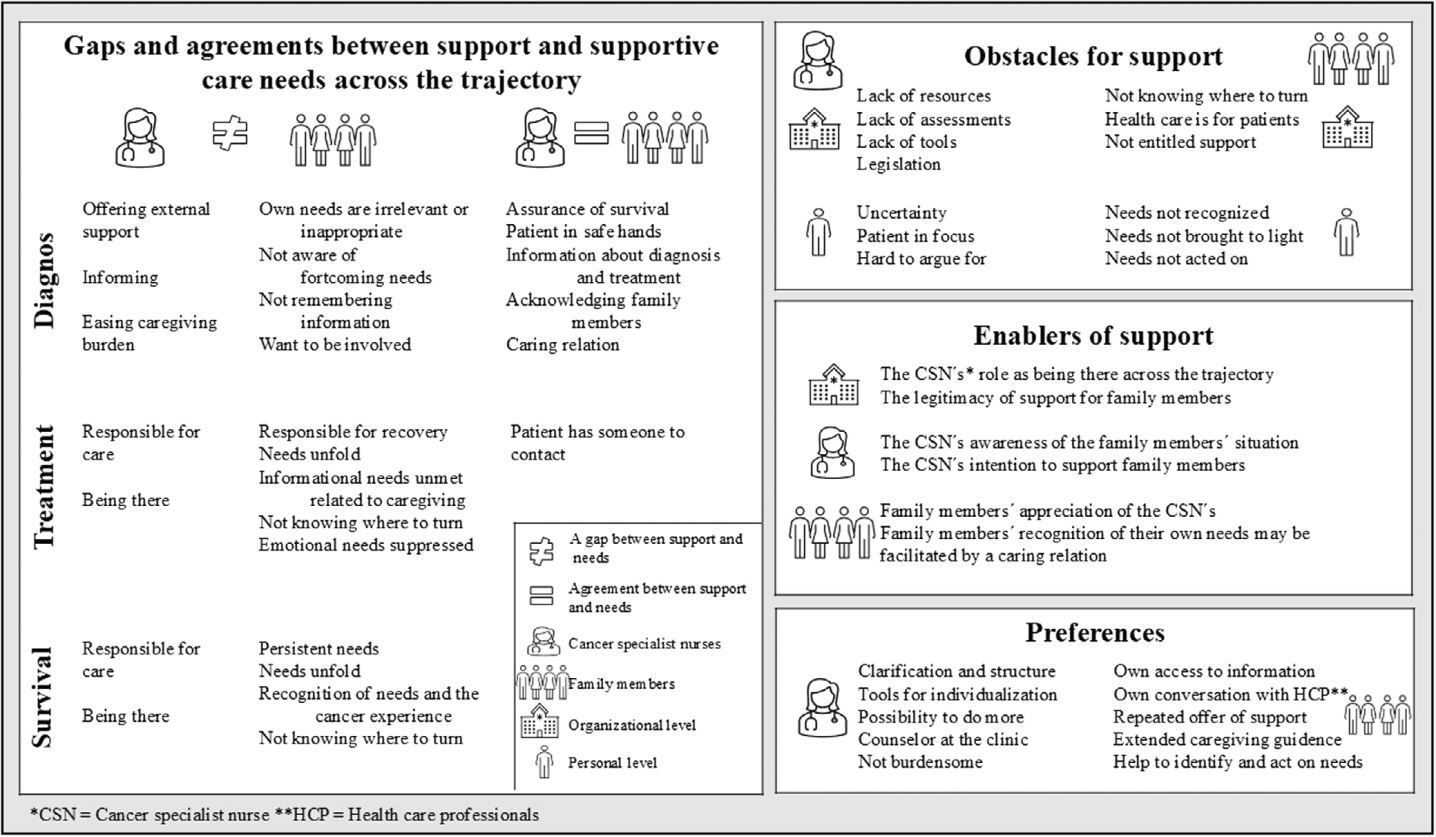
Illustration of the analytical process of the qualitative comparison of the two data sets of family members' and cancer specialist nurses' experiences.

In Step 2, individual semi‐structured interviews were conducted with family members and cancer specialist nurses, following interview guides with open‐ended questions, in accordance with Patton [45]. Interviews with cancer specialist nurses focused on their experiences of, and prerequisites for, supporting family members. Interviews with family members focused on their thoughts about their supportive care needs across the trajectory. These interviews were audio recorded and transcribed verbatim to enable findings to be derived from the participants' narratives [45]. The data collection and the findings are described in detail elsewhere [46, 47]. The two datasets were analysed using qualitative comparative analysis, as described by Lindsay [48], to identify the support preferences of the two groups, gaps and agreements between support and supportive care needs as well as obstacles and enablers on both individual and organisational levels. In addition, the two datasets were analysed to identify both generic and specific needs and support. See Figure 2 for an illustration of the analytical process.

### 3.4 Step 3: Generation of Generic Key Components

In Step 3, generic key components were generated in collaboration with stakeholders through repeated consultations. Consultations were conducted virtually during national workgroup meetings, which allowed for insights from stakeholders from across Sweden and multiple clinical contexts. The key components are elements of central importance for the intended change of an intervention [35]. Prior to the first consultation, the analysis of findings from Steps 1–2 had generated concepts of apparent relevance (e.g., tailoring, proactive, access and suppressing needs). These concepts were presented to the stakeholders in the shape of a ‘mind map’ and they were asked to verbally, in groups, reflect on their content relevance and clinical applicability. Content relevance was supported, yet the need for concretisation was stressed. The concepts were further elaborated upon in two additional consultations, with an analysis of the discussions performed in between, resulting in the following key components: Prepare, Encourage, Enable, Uncover, and Refer.

### 3.5 Step 4: Generation of a Diagnose‐Specific Module Prototype

In Step 4, the operationalisation of the key components into a diagnosis‐specific module prototype was undertaken through consultations with end users from a diagnosis‐specific context (outpatient colorectal cancer care). They were presented with the findings from Steps 1–3 and were asked to elaborate on the clinical applicability of the key components, identifying gaps and agreements as well as obstacles and enablers in relation to the cancer trajectory and the organisation of routine cancer care. Actions (*what*), appropriate supporters (*who*, i.e., nurse, physician, or clinical social worker), and timing of support (*when*) were discussed and finally sketched on a timeline following the routine cancer care process from diagnosis to surveillance.

### 3.6 Step 5: Validation

The key components and their suggested operationalisation (the prototype) were presented to the stakeholders, who were asked for feedback on content relevance and feasibility. Both stakeholders who had been part of developing the model and stakeholders unfamiliar with the development process participated in the feedback event. First, the stakeholders were presented with the development process, steps 1–4, and its findings. Then, the stakeholders were asked to discuss and verbally assess the relevance of the content of (a) the key components and (b) the actions (what, who and when). Then, the stakeholders were asked to discuss and assess the feasibility of the actions (what, who and when). The content relevance of the generic key components and the feasibility of the diagnose‐specific module prototype were supported.

## 4 Results

### 4.1 FamCASP: Families Experiencing Cancer—Support to Prevent Cancer Related‐Illness

**FIGURE 3:**
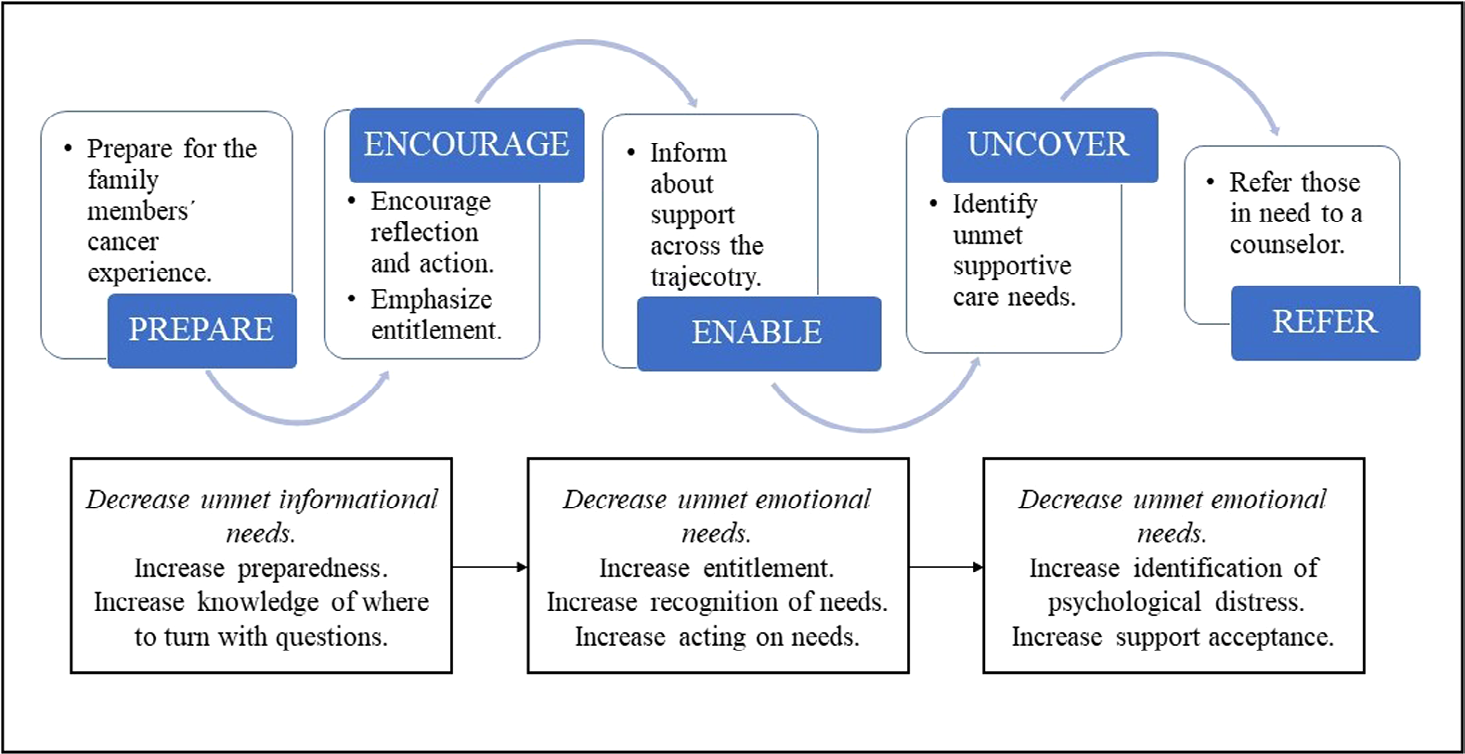
Illustration of the generic key components of the FamCASP. Intended purposes are outlined in boxes.

The FamCASP support model is a generic, nurse‐led, interprofessional model for structured yet individualised support for family members in routine cancer care. The overall intent is to prevent negative health outcomes among family members. The generic model consists of the key components that are believed to apply to family members regardless of age, relationship to the person diagnosed with cancer, cancer diagnosis and setting (Figure 3). These components are intertwined and interact in a process targeting the fact that family members may suppress their needs, something that may act as an obstacle to support acceptance. Hypothesised mechanisms leading to change are, thus, that the support model causes family members to recognise and act on their needs, which will lead to a reduction of unmet needs and thereby prevent a negative impact on family members' health. The key components of the support model are illustrated in Figure 3.

The operationalisation of FamCASP into a diagnosis‐specific module prototype indicated the support model to be adaptable to specific contexts and to diagnosis‐specific needs and prerequisites. These guided the timing and content of supportive actions (diagnosis‐ and treatment‐specific information). Both the main model and the specific module are adaptable to the local context in terms of being applicable in existing systems and allowing for the integration of present support.

### 4.2 Clinical Application of FamCASP

In the colorectal cancer module prototype, at the time of diagnosis, the contact nurse complements existing support with *preparing* family members (verbally and in writing), not only for the illness trajectory but also for the family members' cancer experience: for changing needs, for suppressed needs, for caregiving, and for survival. This includes an awareness that a ‘good prognosis’ may still be a life‐changing event for families and survival may not mean a return to life as it was before. This component aims to lay the foundations for the other components. Contact nurses then *encourage* family members to reflect and act on emerging needs through recognition and confirmation of family members' situation and needs in order to enhance entitlement to support. This includes information about the fact that family members tend to suppress their own needs, in relation to the needs of others and caregiving. This component aims to make family members feel entitled to support and not to suppress their needs, which is believed to help bring to light their needs and thus facilitate individualisation. Then, to *enable* family members to seek support, if needed, verbal and written information about support services across the trajectory is offered. This includes recognition that family members' emotional and informational needs of support may unfold later in the cancer trajectory. This component aims to facilitate family members to act on their needs. Despite these components meeting several needs, family members may hesitate to disclose their needs in order not to be a burden. Hence, helping family members to *uncover* their needs is warranted to prevent cancer‐related illness. This may be eased by strategies promoting communication within the family, by family members having a separate conversation with healthcare professionals, or by using a screening tool to assess family members' needs. This component aims to detect those for whom the previous components are insufficient. Last, *referring* those in need to a counsellor/psychologist, along with information about benefits, based on narratives from family members. This component aims to bridge the gap between psychologically distressed family members and counselling. The previous key components are thought to create enhanced prerequisites for support acceptance.

## 5 Limitation

This study undertook the iterative process of developing a support model to prevent cancer‐related illness among family members through individualised support within routine cancer care involving healthcare professionals, family members, and stakeholders. Throughout its development, local (national) fit was emphasised, which may limit international application. In addition, the support model is designed to enable individualised support, regardless of the sociodemographic characteristics of the support receivers (e.g., age, gender, level of education, religion and ethnicity). However, this approach requires further exploration in the forthcoming evaluations. Moreover, the support model and the prototype are developed to be offered to the families from the time of diagnosis to create prerequisites for family members to recognise and act on their needs across the cancer trajectory. However, the generic support model itself may not cover all support needs at all times. Hence, it is essential to develop diagnosis‐specific prototypes that consider the expected cancer trajectory, cancer stages, and treatments, as well as integrate information about additional support services for families beyond the clinical cancer setting. To this date, only one diagnosis‐specific module has been developed (colorectal cancer). Hence, the applicability of the support model in other contexts needs further exploration.

## 6 Conclusion

Given the global increase of cancer in parallel with burdened health care, prevention of family members' ill health, as a consequence of the cancer experience, should be a priority. Strategies need to be developed that move beyond the evident recognition of the importance of supporting family members into implementable and lasting actions. However, interferences in routine cancer care need to be carefully considered and, ideally, derived from existing structures and support. This project demonstrates how a concurrent consideration of prerequisites for support and family members' needs can generate tailored support models for routine care that are considered relevant, usable, and implementable by healthcare professionals, family members and stakeholders. To facilitate individualisation and uncovering of needs, a digital, interactive screening tool is under development. Evaluations of feasibility and effectiveness will follow prior to implementation. Then, the applicability of the key components in other contexts will be evaluated after having identified their diagnosis‐specific operationalisation.

## Author Contributions

M.S. is responsible for the study design, data acquisition, analysis, manuscript writing, and final approval of the version to be published.

## Funding

The author has nothing to report.

## Ethics Statement

The project was approved by the National Ethical Review Board (Reg. no. 2020–04081; 2021–02601; 2022–02144‐02).

## Conflicts of Interest

The author declares no conflicts of interest.

## Data Availability

The data that support the findings of this study are available on request from the corresponding author. The data are not publicly available due to privacy or ethical restrictions.
